# Musculoskeletal complaints, postural patterns and psychosocial workplace predictors in police officers from an organizational unit of a German federal state police force - a study protocol

**DOI:** 10.1186/s12995-023-00372-8

**Published:** 2023-04-13

**Authors:** Daniela Ohlendorf, Janna Schlenke, Yunes Nazzal, Faiz Dogru, Ioannis Karassavidis, Fabian Holzgreve, Gerhard Oremek, Christian Maurer-Grubinger, David A. Groneberg, Eileen M. Wanke

**Affiliations:** Institute of Occupational, Social and Environmental Medicine, Goethe University Frankfurt, Theodor-Stern-Kai 7, Building 9A, 60596, 60590 Frankfurt, Germany

**Keywords:** Police officers, Germany, Upper body posture, PSQ-Op, Nordic Questionnaire, COPSOQ

## Abstract

**Background:**

Police officers are exposed to a particularly high risk of musculoskeletal disorders (MSDs) and psychosocial stress due to their working conditions. Therefore, the aim of this project will be to assess the occupational physical and mental health of police officers from an organizational unit of the police force of a German federal state.

**Methods:**

The aim is to analyze at least 200 active police officers of a state police force in Germany between the age of 18 and 65 years. In a mixed-methods design, a video raster stereography-based measurement of the upper body posture and a modified version of the Nordic Questionnaire (NQ) will be used for investigating their physical health, while the Copenhagen Psychosocial Questionnaire (COPSOQ) and the Operational Police Stress Questionnaire (PSQ-Op) will be used to analyze their mental health. In addition, job-specific psychosocial factors at the workplace will be assessed (using self-designed questions that were previously evaluated in an expert interview).

**Discussion:**

To date, there is a lack of current questionnaire-based data on the prevalence of MSDs in police officers, or of MSDs associated with injuries or psychosocial workplace factors. Thus, in this study, these MSDs will be correlated with quantitative upper body posture data. If these results prove an increased physical and/or psychosocial stress, then the existing workplace health promotion measures should be analyzed and modified if necessary.

## Introduction

Work-related musculoskeletal disorders (MSDs) are a major problem in the working population and an important determinant of work ability and productivity, with particularly stressful physical and psychosocial work characteristics being predictors of MSDs [1–4]. One occupational group that is particularly at a high risk of MSDs due to the nature of their work is that of police officers [3]. Police officers are exposed to many different challenges and also potentially dangerous situations during their working day [5]. For this work, a good physical, but also mental, condition is essential as the stress situations vary rapidly and the police officers have to adapt accordingly. Police officers are often exposed to high physical stress in their jobs, for example, by wearing heavy body protection suits for several hours, sometimes several days a week [6]. Physical stress may also be a predictor for the development or intensification of complaints in the musculoskeletal system, or can be at least associated with an increased risk of injury. [7]. Hence, it can be assumed that this occupational group is predestined for the development of musculoskeletal diseases, especially in the shoulder-neck region, the back and also the upper and lower extremities [3, 6].

Multi-regional pain has been found to affect 41.3% of Swedish police officers at a frequency of one day per week in the previous three months. [6] The researchers attributed this to wearing mandatory equipment (duty belt, body armor) and prolonged sitting. Furthermore, in Sweden, musculoskeletal disorders are the main reason for incapacity to work among women officers (33%), while for male police officers they are the third cause [8]. Meanwhile, Korean police officers have been shown to experience incidences of pain with occurrences of 44.2% in the shoulder, 41.4% in the lumbar area and 31.2% in the neck [9]. Research involving Polish police officers (in the motorcycle division) who complained of MSDs, revealed that 57.37% of these ailments occurred in the locomotor system, mostly in the lumbosacral (76.17%) and cervical spine (50.93%) areas. These ailments most often appeared after a long ride (53.46%) although they usually disappeared within a few hours of the end of the ride (60.65%) [10]. Police officers in Quebec have also reported of experiencing lower back pain (LBP) in the previous 12 months (67.7%); 96.5% of them linked their LBP totally or partially to their work in the police force [11]. Not only was the prevalence of MSDs found to be very high in the shoulder or back regions, but also the injury rates in police officers [12]. Despite the observations above, it was found that police officers in Minneapolis who engage in higher levels of physical activity are more physically fit, have a lower BMI and experience a lower prevalence of musculoskeletal injuries and chronic pain [13].

Turning to the mental health of police officers, a study of military officers in Rio de Janeiro by Souza et al. [14] revealed an association between psychological distress and other factors such as the ability to react to difficult situations, dissatisfaction with life, health problems (especially digestive, nervous and musculoskeletal symptoms), and adverse working conditions (i.e., excessive workload, constant stress and victimization). Based on these results, they emphasized the need for health promotion interventions (especially for mental health) for the military police.

When gender and age are taken into account, women experience, among other things, greater emotional exhaustion [15] and more stress than men, preferring emotion-focused strategies of coping [16] and appearing to be more resilient [17] than men. Older people have also been found to be more resilient [17]. In addition, women [4] and older people [18] are particularly affected by MSDs. With regard to male and female police officers, it has been found that female police officers experience more burnout [19], emotional exhaustion and stress [19, 20]than men and perceive police stressors differently [21, 22], while regarding the stronger perception of stress in old age, the results diverge among police officers [23, 24]. Queirós et al. [25] conducted an online survey with 1,131 police officers using the PSQ-org. According to this, 88.4% of police officers experienced high operational stress, 87.2% had high organizational stress, 10.9% had critical burnout levels, while 53.8% had low resilient coping. Task-oriented coping was preferred to emotional and avoidance coping. Although women are in the minority in the police, they showed more enthusiasm for work and avoidance coping while the men exhibited more inertia and burnout. Older (professionally experienced) police officers felt more guilty than younger ones and used more emotion-oriented coping, while, in contrast, the younger (professionally experienced) police officers felt less guilty and preferred avoidance-oriented coping strategies.

In Germany, there are findings on the connection between MSDs and psychosocial factors among police officers [3]. In a survey of police officers from the special units of the German police (mainly officers of the special operations command and mobile task forces (German abbreviations: SEK and MEK)) on the influence of psychosocial factors on the development of MSDs (7-day and 12-month prevalence), the occurrence of pain was queried in four regions (neck, shoulder, back, hip) [3]. Thereby, 41.9% of the respondents reported pain in the neck region, 33.8% in the shoulder region, 50.4% in the back and 13.6% in the hip region. For comparison, national data from the Federal Institute for Occupational Safety and Health (BAuA) [26] among others include the 12-month prevalence for MSDs with regard to the working German population. According to this, 48.5% of employees were affected by neck and shoulder pain, 46.3% by lower back pain, 21.4% by knee pain and 15.6% suffered from pain in the hips. Furthermore, von dem Knesebeck et al. [3] found an imbalance between occupational exertion and reward (the Effort-Reward Imbalance Model according to Siegrist [27]), with an approximately twofold increased risk of suffering from neck, back or hip complaints. This was observed regardless of age, gender, social status, unit affiliation or physical workloads [3]. The health monitoring of Berlin police officers (n = 573) comprised various categories, such as musculoskeletal complaints, work organization-specific requirements, police-specific immanent activity requirements, conflict between work and private life or gratification crisis [28]. The results reveal a 12-month prevalence of back pain at 41% and of shoulder/neck pain at 43.5% among the police officers[28]. The Effort-Reward Imbalance Quotient for police officers was 86% above the value of 1, thus, they were highly exhausted (> 1 = negative gratification ratio). This was particularly the case for men and older or senior officers. Furthermore, the researchers found a significant difference between different organizational units in this context. Dimensions such as expenditure, esteem, professional development, consistency of the work situation and willingness have been found to be significant predictors of work-related exhaustion and the subjective probability of health-related early retirement [29]. These factors are influenced by other variables such as age, chronic illness, involvement in work or place of employment [29].

In general, the demands on German police officers have increased considerably in recent years [5], and these have been accompanied by an increase in musculoskeletal complaints [28] and negative psychosocial effects on job satisfaction [3, 28].

Furthermore, police work often involves manual work or physical strains that can cause musculoskeletal disorders. As these are occupational activities, they are referred to as work-related musculoskeletal disorders; these include forced posture (i.e., remaining in a given position), a restricted range of motion over a long period of time, prolonged static positions, asymmetrical, one-sided or repetitive activities, carrying heavy loads or not changing loads, prolonged forward bending or trunk rotation, and working with hands above shoulder height [30]. These situations can occur, for example, during demonstrations when the police officers have to wait for several hours in a confined space (police car or van) together with several colleagues wearing complete protective gear with a weight of 13 kg (for example, an upper body protector (size L) 7.5 kg, arm protectors and leg protectors 3 kg, helmet 2.5 kg; weight depending on body size approx. + 4.7 kg with each size) and where they have to remain static with restricted movement, or when they have to remain standing in full equipment at a given location for several hours afterwards. In summary, all movements are restricted by using this protective gear. Muscular imbalances can also develop from these occupational activities which may manifest themselves in the upper body posture. However, there is a general lack of proven evidence on the exposure-response relationship between occupational risk factors and musculoskeletal disorders. In particular, this refers to the relationship between subjective questionnaire-related data and quantitative data. In their meta-analysis, Nordana et al. [31] found quantitative exposure-response relationships between the physical workload of the neck and arms and musculoskeletal disorders of the neck and shoulders. This has been demonstrated on the one hand with the Nordic Questionnaire and, on the other hand, with physical examinations (such as electrogoniometry or electromyography).

Following Nordana et al. [31], the question now arises whether these relationships are also the case for police officers of an organizational unit from a German federal state police force. Since these police officers often wear a protective suit, it seems reasonable to assume that MSDs can develop from this and that these can be observed in the upper body posture. Video raster stereography is a technique that is already used to measure the upper body posture [32–34]. In addition, there are available current standard values depending on age, sex and BMI that can be used as reference values [32].

Knowledge of the existence and extent of occupational musculoskeletal complaints or of postural patterns, or the stressful psychosocial factors at the workplace and stress factors is extremely important in order to identify approaches for effective behavioral and/or relationship-based preventive measures. It is clear from the available studies that, to date, there exists predominantly questionnaire-based data on the prevalence of MSDs or MSDs associated with injuries or job satisfaction [1–3, 8], however, correlations of this data with quantitative data, such as the upper body posture, are lacking for police officers in Germany. In particular, with regard to the prevalence of MSDs and psychosocial workplace predictors, it would be interesting to know whether there are differences between men and women.

## Aims

Therefore, the aim of the present project is to assess the occupational physical and mental health of police officers from an organizational unit of a German federal state police force, within the framework of a mixed-methods design. Physical health will be assessed by means of a three-dimensional measurement of the upper body posture via video raster stereography (quantitative data) and the Nordic Questionnaire (NQ). The NQ records the musculoskeletal complaints of each body region in terms of a 7-day and 12-month prevalence based on subjective assessment. For the mental health assessment, the Copenhagen Psychosocial Questionnaire (COPSOQ) and the Operational Police Stress Questionnaire (PSQ-Op) will be used. These questions can be categorized into the following dimensions: physical and mental job demands, job control and development potentialities, work-life-balance, relationship to colleagues and superiors, workplace factors and job satisfaction.

Therefore, the following overarching hypotheses will be tested in this project:

### Hypothesis 1

Police officers show a higher prevalence of MSDs in comparison to the general population.

### Hypothesis 2

Higher psychosocial stress factors correlate positively with a higher risk of MSDs.

### Hypothesis 3

Higher values of the PSQ-Op correlate positively with an increased level of psychosocial stress factors.

### Hypothesis 4

There are differences in all three questionnaires (NQ, PSQ-Op, COPSOQ) between the male and female respondents.

### Hypothesis 5

Particularly pronounced complaints in the shoulder-neck region and the back are assumed to be reflected in an asymmetrical upper body posture.

### Hypothesis 6

The prevalence of musculoskeletal complaints correlates positively with age.

## Materials and methods

### Subjects

At least 200 police officers from a single, federal state police force in Germany are to participate voluntarily in this study. The test persons should be between 18 and 65 years old and work in active service. Since the police service is an extremely diverse profession, with different work demands depending on the department, the planned study focuses on police officers who have a minimum level of physical ability to cope with the given working conditions, for example, wearing up to 20 kg of body protection gear on duty or being routinely exposed to physical confrontations.

Therefore, the subjects will only be included in the study if they are not impaired in terms of performance on the day of the measurement. Prevalent, known musculoskeletal conditions that do not limit the current occupational performance within the units under study do not constitute exclusion criteria. However, excluded from the study would be those police officers who have momentary or permanent occupational performance impairments that do not permit participation in active duty as evidenced, for example, by a certificate of incapacity for work.

An approved ethics application from the Department of Psychology and Sports Science at the Goethe University Frankfurt (No.: 2022-07) has been submitted for the conduct of the study.

### Recruitment

The program will be promoted via in-house e-mails from the health department of the respective company and, at the same time, the possibility of registering for the study will be offered. In order to increase the willingness to participate and to clarify any questions in advance, meetings will also be organized with the respective department heads before the start of the studies. An information sheet, which will be posted in the respective departments, will provide additional information about the course of the planned study and the possibility to participate.

### Questionnaire survey

The online questionnaire is to be implemented via the SoSci-Survey portal and, thus, made accessible to every participant. The questionnaire is composed of questions from three areas.

Firstly, a modified version of the Nordic Questionnaire (NQ) will be used. This questionnaire was developed by Kuorika et al. [43] in 1987 as part of a project funded by the Nordic Council of Ministers. The questionnaire to be used in this study is based on a reformulated edition of the Federal Institute for Occupational Safety and Health (BAuA) and is currently still being tested. The BAuAquestionnaire differs from the original version by Kuorinka et al. (1987) in that the questionnaire logic has been changed and an additional body region (the lower leg) has been subdivided. However, the questionnaire on which this work is based is oriented towards the original nine body regions designated in the version of the Federal Institute for Occupational Safety and Health (BAuA) and included in the questionnaire translated into German [44]. Corresponding illustrations are also used from the BAuA version. However, some of the questions deleted by Liebers et al. (2021) from the BAuA modified version have been reinstated for the questionnaire used in the present study’s questionnaire. The questionnaire also asks about functional limitations, whether a physician has been consulted within the last 12 months and whether sick leave has been taken due to existing complaints, for example, in addition to the 12-month and 7-day prevalence of MSDs in the relevant body regions [45]. Current studies in the field of occupational medicine and ergonomics use numerical rating scales in the examination of musculoskeletal pain [35, 36]. Therefore, a modified version of the NQ, supplemented by a numerical rating scale, will also be used in the planned examination. The numerical rating scale (NRS) (0–10) rates the severity of the complaints to the individual body regions. A scale of 0 means no pain while a score of 10 means worst pain imaginable.

This makes it additionally possible to differentiate the disorders with regard to the intensity of the complaints. Furthermore, the officers interviewed should be given the opportunity to express an assumption about the cause of the complaints within a free text field, for example, due to the body protection equipment worn on duty. The validity, reliability and sensitivity of the NRS to changes in pain have been rated as high [46–48].

Secondly, the questionnaire contains the German version of the Copenhagen Psychosocial Questionnaire (COPSOQ), a self-assessment questionnaire for recording psychosocial factors at the workplace. The COPSOQ was first presented and successfully used in Denmark by Kristensen et al. [49] in 2005; it has also been classified as valid and reliable and is independent of language. The validity and reliability also applies to the German version [50] which has been shortened for the planned survey due to time constraints (questions that were too unspecific for the police service were deleted). Thus, the German version corresponds to the recommendation of Nübling et al. [51] who first examined the translated version of the Danish original in Germany in 2006. The COPSOQ has already been used with various occupational groups such as teachers [37], nurses [38], researchers in mental health [39] and the general Dutch working-age population (n = 55,950) [40].

In this questionnaire, both the work-related stresses/strains and complaints are recorded. Different dimensions are surveyed here, such as the demands of work (quantitative, emotional, work-privacy conflicts), the influence and development opportunities, other work-related factors (work environment, insecurity and conditions), social relations and leadership (feedback, predictability, leadership quality, role clarity, role conflict, sense of community, support at work), as well as the impact of the former dimensions (job satisfaction and engagement, general health, burnout symptoms, presenteeism). The COPSOQ contains response categories ranging from “to a very high extent” to “to a very low extent,“ and the description of the frequency from “always” to “never/almost never”. A high score implies a high degree of the assessed condition.

[49][50][51]The Operational Police Stress Questionnaire (PSQ-Op) is a psychometrically based questionnaire that is used to measure the stress factors of police work [41, 42]. This was developed by McCreary and Thompson in 2006 and is a psychometrically based questionnaire to measure the stress factors involved in police work [41, 42, 52, 53]. The original 20-item questionnaire has been shortened to five relevant questions for this study, with a numerical rating scale (0–7; 0 = no stress at all, 7 = a lot of stress) offered here as response options. Thus, the version to be used in this study is a translation of the Operational Police Stress Questionnaire.

Furthermore, ten questions related to the sample group and four general questions related to the person will be used. In addition, an expert interview consisting of experienced male and female police officers,, as well as newcomers to the profession, will be conducted for the purpose of occupation-specific adaptation. Questions related to the subject collective refer to questions concerning the body protection equipment, the side of the body and the localization (hip/thigh) of the service weapon carried. Moreover, the present sporting activity of the police officers (professional and recreational) are to be recorded. In the same way, the recording of the individual’s state of health is to be taken into account by indicating any previous musculoskeletal disorders and service injuries.

The questionnaire will be created online via the SoSci Survey server. A pretest on students of the police academy will be carried out to check the practicability and quality of the questionnaire. In the pretest, in addition to the regular answering of the questionnaire, comments on the individual pages should be possible in order to note remarks and any difficulties in understanding. Based on the pretest, the questionnaire will be adapted if necessary.

### Three-dimensional back scan

The Bodymapper (ABW GmbH, Frickenhausen, Germany) will be used for the three-dimensional measurement of the dorsal upper body posture. Using video raster stereography, the back is optically recorded at a frequency of 50 Hz and a resolution of 1/100 mm at a distance of 90 cm. The system error is specified as < 1 mm (manufacturer’s specification) and the reproducibility is limited (< 0.5 mm) by the calculations of the upper body posture which is defined by markers (retroreflective stickers) directly on the skin. The intra- and inter-reliability of this measurement system is described as good [54]. Furthermore, Yi et al. [54] have also demonstrated a correlation between the Cobb angle (by using radiography) and the lordosis and kyphosis angles (by using the body mapper). However, the accuracy of the data was found to increase with the experience of the examiner who placed the six predefined landmarks (cervical vertebra C7, rima ani, caudal apex of the left and right scapula, and the spina iliaca posterior superior anterior left and right) on the back of the person being measured. Therefore, an experienced examiner will be used for this study.

**Fig. 1:**
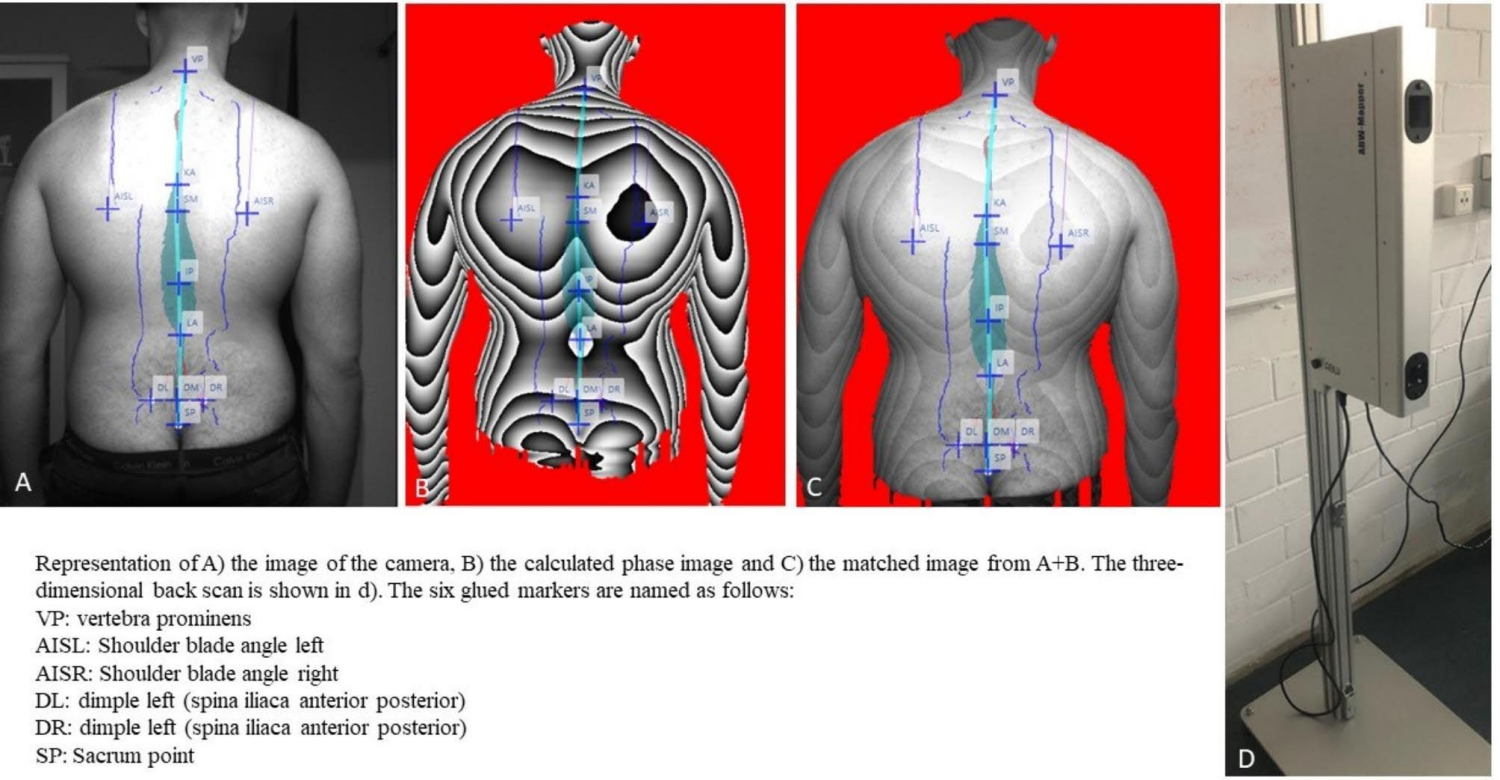
Representation of (A) the image of the camera, (B) the calculated phase image and (C) the matched image from A + B. The three-dimensional back scan is shown in d)

Finally, twenty-three evaluation parameters to be used in the proposed study have been grouped into three sections: the shoulder, spine and pelvis area (Fig. 1). A detailed description of the parameters can be found in the methods paper of Ohlendorf et al. [32, 55].

### Measurement protocol

Prior to the examination, each participant will generate a four-digit code which is used both at the beginning of the questionnaire survey and for the three-dimensional back measurements in order to record the subject data pseudonymously.

Both series of measurements should take place within one hour on the same day. For the back measurement, three measurement repetitions will be carried out that will be averaged for the statistical evaluation.

All collected data will then be transferred to the statistical program SPSS and saved anonymously as a data set.

### Statistical data analysis

Only those questionnaires that have been fully completed will be included in the questionnaire’s evaluation. The data formatting will be carried out via Excel. The questionnaires will be checked for plausibility in the answering behavior. The back measurement data will also be checked for measurement errors and only complete data sets will be included in the evaluation. All data will be evaluated in SPSS Statistics 26 (IBM Deutschland GmbH, Ehningen, Germany).

For the evaluation of the results, the data will be tested for normal distribution while descriptive procedures for sample characterization will be applied and calculated for all variables, taking socio-demographic characteristics into account. The respective response categories are coded into numerical values, based on the assumption that the characteristics are measured metrically, even though the data are ordinally scaled. This is a common procedure in research practice and is much discussed in the literature; it is based on a scale-theoretical assumption that may be accepted if the content hypothesis is empirically confirmed [56]. Depending on the outcome, parametric tests for normal distribution will be used or pseudo normalization will be achieved by a rank transformation of the data. In addition, methods of interference statistics as well as correlation calculations are to be applied. The significance level will be set at 5%. Single items which collect psychosocial workloads factors will be summarized to a new variable in analogy to the dimensions defined above. Responses for each item will be oriented so that higher values implicate a higher psychosocial workload related to the respective dimension. Cronbach’s Alpha will be applied to measure internal consistency of the new index. Based on this, logistic regression will be performed, while the models will be adjusted for important confounders such as age, sex and the extent of physical exercise/sport. A Bonferroni correction will be applied to counteract multi comparison effects.

#### Hypothesis 1

The overall MSD score and the 15 MSD scores per zone are compared with the MSD scores of the general German population [4]. To counteract differences in the gender distribution, the test will be performed for both sexes individually.

#### Hypothesis 2

A Pearson correlation will be used to determine the correlation between the 9 dimensions of the three questionnaires and the MSD scores.

#### Hypothesis 3

A Pearson correlation between the PSQ-Op and dimensions of the NQ and COPSOQ will be performed.

#### Hypothesis 4

A MANOVA test will be performed between the three questionnaires with respect to gender.

#### Hypothesis 5

A Pearson correlation between the MSD score for the shoulder and neck and the upper body asymmetry value derived from the video raster stereography is calculated to test the relationship between the MSD score and upper body posture.

#### Hypothesis 6

A Pearson correlation between age and MSD score will be conducted.

## Discussion

According to the current state of knowledge, there are only a few studies in Germany [3, 28] that have dealt with occupation-specific musculoskeletal complaints in police officers. Musculoskeletal disorders are a very heterogeneous group of symptoms and diseases to which no specific pathophysiological mechanisms can often be attributed [57]. The demands on police officers in Germany have increased considerably in recent years [5], and with them, the psychosocial workplace situation as well as physical stress [28]: 39% of the respondents of a Berlin police department stated that it was rather likely or even very likely that they would have to retire early for health reasons [58]. These results once again imply the need for a scientifically based study of the mental and physical health of police officers as a basis for the targeted implementation of preventive measures.

The analysis of occupational MSDs or posture patterns, in combination with stressful psychosocial factors at the workplace or stress factors, provide important insights into the overall workplace situation of the physical and mental health of police officers. From this, behavioral and/or relationship preventive measures can be derived to preserve or maintain the health of the employees in order to prevent long-term consequences that may include reduced physical resilience, reduced well-being, a reduced ability to cope sufficiently with everyday activities or a high number of days of incapacity to work, up to and including early retirement. It should also be borne in mind that musculoskeletal complaints are associated with chronic pain and can result in physical, functional impairment that can also have an indirect negative impact on occupational productivity. The earlier in working life that such work-related complaints occur, the higher are the health costs incurred during the years of occupational work to the time of retirement.

The goal of generating scientific data in a mixed-methods design, as aimed in this project, is to counteract the problems described above and, thus, reduce the incidence of job dissatisfaction, absenteeism or even occupational disability. In this way, existing data for German police officers regarding MSDs and job satisfaction [3, 28] could be tested and expanded by using the Copenhagen Psychosocial Questionnaire and the Operational Police Stress Questionnaire, as well as the biomechanical measurement of upper body statics in conjunction with data from the Nordic Questionnaire.

Based on the available results, it would be plausible, with regard to a potential increased psychological stress, to reduce the stress in a targeted manner, for example, through an external psychological counseling officer, the development of a group training for better handling of increased work demands or through targeted management training towards a more employee-oriented management style [58]. This can be duly facilitated because the questions, which have a psychosocial focus among the state police officers, specifically ask about aspects such as emotional stress and job satisfaction, but also about the influence of these aspects on their decisions.

If the survey of the prevalence of MSDs in combination with the recording of upper body posture reveals that there is increased stress in certain parts of the body, the existing measures for workplace health promotion could be analyzed and modified if necessary. For other occupational groups, such as office workers [59], hospital staff [60] or men working in military service [61], targeted strength training has been identified as a positive method. Systematic stretching training or targeted yoga exercises have also been shown to have positive effects [62]. In addition, there are work-related individual preventive approaches, such as the Back College of the German Social Accident Insurance Institution for Health and Welfare Services (German abbreviation: BGW) for the prevention of back pain among nursing staff, or the Knee College of the German Social Accident Insurance Institution for the Construction Industry (German abbreviation: BG Bau) [63, 64].

Both programs endeavour to take into account the complexity and multi-causality regarding the development of musculoskeletal complaints by following a more holistic approach. Thus, in addition to physiotherapeutic physical and sports medicine therapy, the 3-week Back College (BGW program) provides occupation-specific exercise as well as psychological counseling with regard to dealing with pain and stress. Other components include educational lectures, nutritional counseling and an aftercare program. Overall, significant reductions in pain can be achieved with the help of the program [63]. The BG Bau Knee College, on the other hand, is intended for a longer period of time and, in addition to a 3-week build-up phase, includes an alternation of a total of two 12-month training phases (with an attachment to a fitness studio close to home) as well as a respective refresher phase following the training phase. This program also seems to be effective in improving or preventing knee joint complaints by significantly increasing the maximum strength of the thigh muscles and the gait asymmetries, as illustrated by an interim evaluation after 12 months [64].

### Limitations

Methodological limitations result from the chosen study design (a cross-sectional study) which, apart from the three-dimensional back measurement, is based on a subjective assessment. Objectively measurable data that are generated result from the three-dimensional back measurement. However, causal conclusions cannot be drawn from this although comparisons with reference values already collected are possible. Furthermore, the subjective survey of MSDs and psychosocial work factors is a momentary survey that depends on the current state of the day. An additional clinical examination, which would supplement the data from a medical perspective with an objective finding, is not planned due to time and organizational reasons, thus, the survey of MSDs will only be based on subjective statements.

Furthermore, MSDs are an extremely heterogeneous, complex collection of a wide variety of symptoms at the center of which are pain, loss of function, degenerative structural changes and psychological factors, and which are also characterized by a multi-causal genesis [65]. The manifestation of MSDs with the same workload can sometimes be completely different due to the individual’s training and performance conditions, different stress perceptions and resistance as well as other environmental factors [65]. Women [4] and older people [18] are particularly affected by MSDs - both groups that are presumably hyphenated in the sample to be studied. At the same time, this makes it difficult to compare gender and age because the sample size is too small in the case of suspected hyphenated groups.

Moreover, only queries on whether, how often and to what extent complaints occur in the different regions will be surveyed; no allocation to specific activities can be obtained from these questions. An activity-specific analysis in the sense of an ergonomic assessment, as well as the identification of activities that represent particularly high physical strains, is, therefore, a future research question.

## Conclusion

Knowledge concerning the existence and extent of occupational musculoskeletal complaints of police officers is of utmost importance in order to identify approaches for effective occupational-specific behavioral and/or proportional preventive measures. The implementation of measures based on scientific data can help to reduce the incidence of musculoskeletal complaints and, thus, reduce the incidence of absences from work or occupational disability in the long term.

## Data Availability

All data generated or analyzed during this study will be included in the published article.
